# Air, Air, Air: a champion midwife programme in Tanzania using HOT neonatal resuscitation—lessons learned

**DOI:** 10.1093/trstmh/trab154

**Published:** 2021-10-06

**Authors:** Jan Becker, Chiung-Jung (Jo) Wu, Chase Becker, James Moir, Marion Gray, Meshak Shimwela, Florin Oprescu

**Affiliations:** Midwife Vision Global Ltd, Tanzania, Senior Clinical Midwife, Director; University of the Sunshine Coast, QLD, Australia; Member in the General Division of the Order of Australia; University of the Sunshine Coast, 1 Moreton Bay Parade, Petrie QLD 4502, Australia; University of Nicosia, Medical School, Makedonitissis 46, Nicosia 2417 Cyprus; Suite 1, Nucleus Medical Suites, 23 Elsa Wilson Drive, Buderim QLD 4556, Australia; University of the Southern Queensland, 37 Sinnathamby Blvd, Springfield Central QLD 4300, Australia; Temeke Regional Referral Hospital, Temeke Road Adjacent Sterio market, Dar es Salaam, United Republic of Tanzania; University of Sunshine Coast, 90 Sippy Downs Dr, Sippy Downs QLD 4556, Australia

**Keywords:** collective self-efficacy, midwife, neonatal resuscitation, training, transferring clinical skills

## Abstract

**Background:**

Tanzania has approximately 40 000 newborn deaths per year, with >25% of these linked to intrapartum-related hypoxia. The Helping Babies Breathe^©^ and Golden minute^©^ (HBB^©^) programme was developed to teach skilled intervention for non-breathing neonates at birth. While Helping Babies Breathe^©^ and Golden minute^©^, providing training in simulated bag and mask ventilation, is theoretically successful in the classroom, it often fails to transfer to clinical practice without further support. Furthermore, the proclivity of midwives to suction excessively as a first-line intervention is an ingrained behaviour that delays ventilation, contributing to very early neonatal deaths.

**Methods:**

The ‘champion’ programme provided guided instruction during a real-life resuscitation. The site was Amana Hospital, Tanzania. The labour ward conducts 13 500 deliveries annually, most of which are managed by midwives. Brief mannikin simulation practice was held two to three times a week followed by bedside hands-on training (HOT) of bag and mask skills and problem solving while reinforcing the mantra of ‘air, air, air’ as the first-line intervention during a real-life emergency.

**Results:**

Champion midwives (trainers) guided instructions given during a real emergency at the bedside caused learners beliefs to change. Trainees observed changes in baby skin colour and the onset of spontaneous breathing after effective ventilation.

**Conclusions:**

Visible success during an actual real-life emergency created confidence, mastery and collective self-efficacy.

## Introduction

Tanzania has approximately 40 000 newborn deaths per year,^1^ with >25% of these deaths linked to intrapartum-related hypoxia.^2^ The Helping Babies Breathe (HBB) programme was developed to address this problem. Tanzania was part of the global rollout in 2009 and saw a significant 47% reduction in very early neonatal deaths (VENDs) within 24 h.^3^ However, simulated bag and mask ventilation (BMV) skills have failed to transfer to clinical practice.^4^ Furthermore, the proclivity of midwives to excessively deeply suction the baby's oropharynx is an ingrained behaviour that delays ventilation, contributing to VENDs. The problem was that despite passing traditional HBB training, the transfer of bag and mask clinical skills was not evident. Immediate return to the use of suction as the first-line intervention for a non-breathing neonate was a barrier that was challenging to overcome.

**Figure 1.**
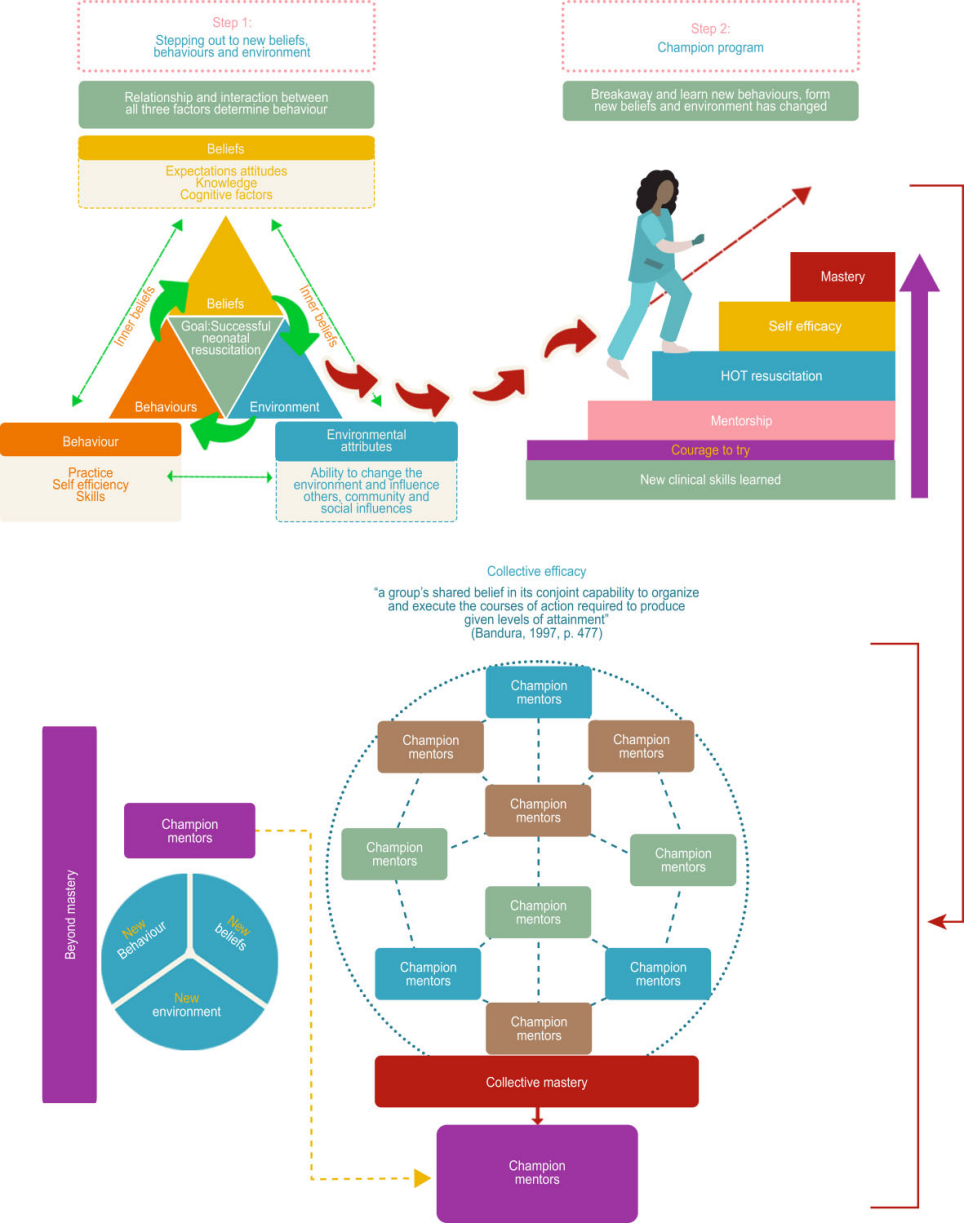
Becoming a champion midwife based on Bandura Social Cognitive Theory.

The ‘champion’ midwife programme (Figure 1) was developed at Amana Hospital, Tanzania to train midwives in effective skilled neonatal resuscitation. The key components were hands-on training (HOT) during a real-life emergency resuscitation on a non-breathing baby in the labour ward with a skilled champion instructor at the bedside after brief BMV skills instruction on a mannikin. The mantra of ‘air, air, air’ rather than suction as the first-line intervention was reinforced in the classroom and in the ward. Eight local midwives were trained as champions and carried out HOT resuscitation training at the bedside during the study period. A training report in August 2021 highlighted that this training is being delivered two to three times per week by local champion midwives, suggesting the long-term sustainability of the project.

The aim of this research was to explore what empowers midwives to perform skilled neonatal resuscitation in Tanzania. The overarching aim was to save the lives of babies who fail to breathe at birth.

## Methods

### Setting

The study was performed at Amana Hospital in Tanzania, with a catchment area of approximately 1 million, plus outlying hospitals that refer patients. Midwives manage the majority of the 13 500 deliveries annually. Often the workload is overwhelming and the midwives **neonatal resuscitation** skills are limited.

Resources and equipment vary. On many days, electricity, running water and hand soap are not reliably available. There is a limited or nil supply of sterile gloves and basic medications, which is a problem in many facilities in sub-Saharan Africa.

### Problem

Upon entering the labour ward during a morning shift in 2017, authors JB and CB saw six babies who had died overnight on a bench labelled ‘dead baby’. This was the catalyst for creating solutions to reduce VENDs.

JB and CB observed that despite passing the HBB assessments, within minutes of re-entering the labour ward, midwives reverted to prolonged deep suctioning for birth asphyxiated babies. This propensity to be engrossed in deep suctioning appeared to be so ingrained as a core practice that despite updated training and skills, most midwives rarely utilized BMV. When they did, it was too late to assist the baby in breathing or having any chance of survival.^3^

Mastery of BMV skills after HBB training on mannikins did not translate into clinical practice.^3^ The midwives’ resuscitation skills were inadequate or were not used at all, despite passing both the written and practical simulation tests. Current and initial research on the HBB programme in sub-Saharan Africa highlighted the difficulty of transferring simulated neonatal BMV skills into clinical practice.^4^ However, some studies have utilized low-dose, high-frequency mannikin training drill practice, the formal appointment of mentors who teach simulation sessions, the development of mannikin training guides and structured on-the-job training, master trainer mentoring via phone and e-mail and peer-to-peer learning. One study in Rwanda cited clinical mentorship as a critical element in the reduction in birth-asphyxiated neonates being admitted to the neonatal unit.^5^ However, no studies have utilized bedside HOT resuscitation to guide clinical skills mastery, the key element of the champion programme.

### Solution

The champion programme was developed based on Bandura's Social Cognitive Theory (see Figure 1), with the cornerstone of the programme being integrated practical HOT during a real-life emergency neonatal resuscitation and troubleshooting, fully supported at the beside by a skilled instructor. The intervention was developed to create or enhance self-efficacy, with the goal of empowering midwives to perform an appropriate resuscitation and achieve BMV mastery.

To engage local midwives, the solution had to be clinically and culturally relevant. The labour ward instruction is frequent (two to three times per week) to capture the fundamentals in a short timeframe (5–15 min) at opportune times for midwives to practise BMV skills on a mannikin. The availability of these materials has helped reduce stress and nervousness, as individual learning needs are integrated into hands-on resuscitation.

The champion midwife (trainer) is a vital element that enables real-time, reinforced practical skills transfer and common resuscitation troubleshooting. Time-critical, clinically based, hands-on instruction builds belief, confidence and mastery. The mentor utilizes verbal persuasion and vicarious experience while providing the trainee with the tools to manage emotional arousal.

The training goal was shifted from a focus on the trainee to a faithful concentration on the neonate's ventilation within the golden minute (see Table 1).

**Table 1.** Elements of the champion programme based on Social Cognitive Theory^6^

Session	Approach	Social Cognitive Theory element/area
Session 1	Introducing the champion programme: a clinically skilled mentor guides and instructs the trainee through hands-on training (HOT)^1^ during a real-life emergency neonatal resuscitation conducted by the mentor	Verbal persuasion
Classroom or in the labour ward at the mannikin setup area (cold education) (1–5 min)	Verbally convey the important points of successful resuscitation: an unequivocal focus on the golden minute to help the baby breathe; warm, dry, stimulate; and bag and mask—air, air, air. Provide the trainee with the HBB second edition workbook in the preferred language. At each session, highlight areas in the workbook to review at home.	
Session 2	Simulation of clinical skills with a mentor (skilled midwife) demonstrating the use of bag and mask and basic resuscitation on a mannikin set up in the labour ward providing opportune training when there is a break in deliveries.	Vicarious experience
Clinical skills (5–10 min)	Highlight and repeat at every session: prepare the bag and mask for every delivery; initial actions—warm, dry, stimulate; and if not breathing, then BMV—air, air, air. Follow the demonstration with analysis and discussion with the midwife, creating a positive interplay for open discussion	Mastery
Session 3	The labour ward after watching a mentor during HOT resuscitation	Self-appraisal of psychological state
Transfer of clinical skills (5 min) (HOT education)	Having a trainee stand beside the champion midwife to see first-hand the changes in the baby's colour, tone and breathing. Guiding the midwife through the impact of her own feelings and thoughts. Highlighting the emotional connection and identifying feelings of anxiousness, panic and fear. Assisting the midwife to gain insight into the trainees skills and learning modalities. Instilling the courage to try.	Identifying responses and emotional reactions
Session 4	In the labour ward, the midwife resuscitates with BMV	Verbal persuasion
Clinical skills in the labour ward	Provide feedback and debriefing after HOT during a real-life neonatal emergency resuscitation in the labour ward with BMV	Vicarious experience
Repeat up to four or five times (1–5 min)	Expanding the trainee's knowledge for mastery of BMV: foetal heart monitoring and use of the partogram to assess labour, delay cord clamping, care for thermoregulation, infection prevention measures and immediate breastfeeding if the baby is breathing normally after resuscitation	Self-appraisal, emotional arousal, which assists the cognitive phase, and mastery
Session 5 (5–10 min)	Biweekly BMV skills in the labour ward taught by the mentor on a mannikin, with clinical skills transfer being monitored by the mentor.	Mastery vicarious experience

^1^HOT resuscitation is hands-on training during a real-life neonatal resuscitation emergency with the guided and full support of a clinically skilled midwife (champion) to instruct, encourage and provide real-time troubleshooting and hands-on support. HOT also implies the emotional arousal, urgency and intensity of both the training and the situation managing a non-breathing floppy blue baby who requires immediate focused skilled intervention. HOT resuscitation is more than mentorship. It is a deep-seated commitment made by the champion midwife and trainee to the golden minute, to the neonate and to the skills required to successfully care for a non-breathing neonate at birth. The totally dedicated champion is there to pass on clinical skills to the trainees and to build confidence and hope.

Seasonal differences do not appear to affect the champion programme; however, additional training, which covers topics that complement the champion programme and address maternal factors that can affect neonatal outcomes is held weekly and intrapartum-related hypoxia may impact outcomes outside the champion programme.

One of the programme's significant aspects is the emphasis on and reinforcement of the fact that the midwives are the baby's only ‘champion’. The midwife pledges to be resolute and to concentrate on the baby for 1 min at delivery.

Mentorship, another component in this study, consists of ongoing support to champion midwives using e-mail and WhatsApp via smartphones that has aided in maintaining momentum and a long-term commitment to the sustainability of this programme (see Table 2).

**Table 2.** Key lessons learned from HOT resuscitation training

Key lessons learned
1. Teaching and learning need to be moved from the training room to the bedside, where seeing real success (baby alive after resuscitation) builds self-confidence in midwives and creates further champions.
2. There must be a focus on unlearning harmful techniques that may be ingrained in daily practice. Doing so is not an easy process. However, it is doable but requires persistence.
3. Mentors can create frequent opportunities for immediate feedback by focusing on BMV as the first-line intervention after stimulation (positive changes in terms of confidence can be seen). Mentors guide the trainees through live (HOT resuscitation) real-time troubleshooting.

The long-term relationships formed by the original champion midwives, JB and CB, by working together with local midwives for many years have created a platform of trust, accountability and hope. Furthermore, a sustained commitment to mentoring by JB and CB to the champions who deliver this programme appears to have created collective self-efficacy and pride in being part of preventing stillbirths and VENDs.

### Sources of information and data

Evaluation data were collected via observations and semi-structured interviews (from July 2019 to August 2020) with midwives who had worked in the labour ward for a minimum of 6 months full-time, had experienced VENDs, had completed the champion programme and had conducted a minimum of three neonatal resuscitations. The researchers documented insights into the champion programme and midwife empowerment. As of 2021, the programme continues with champion midwives delivering training and providing guidance for new midwives and supervision of HOT resuscitation.

## Results

Approximately 62% (n=13) of the original midwives in the labour ward became the initial champions. The change in suctioning habits was the most difficult to teach. One midwife expressed the issue as follows:

‘Past years, I'm always using this suction tube to suck and suck and suck and suck, but nowadays, I rarely suck, and now, in my labour ward, I have not even had a suction machine for almost one year, and it is not affecting me.’ —Mkunga_02

HOT in the labour ward was regarded positively. It was the most influential component of the champion training.

‘On-the-job training is more effective than classroom. Because you teach them in a class once a week and when they come back to the labour ward you get the baby who needs resuscitation, you can teach them through live baby’. — Mkunga_02

They observed a change from blue to pink in the skin colour of the baby. This feedback increased their confidence and self-efficacy and transformed the participating midwives into champions.

### Clinical outcomes

An excerpt from the training report highlights the clinical implications of HOT training.

‘For the staff, they are now up-to-date on the use of bag and mask rather than the penguin and on advanced resuscitation’.

‘We have better outcomes, as we now use bedside resuscitation, and we shout to prepare for resuscitation before delivery’.

‘Training is three to four times a week, and other days, mentorship is conducted. On each shift, no one needs to have an asphyxiated baby.’ —Champion Training Report July 2021

Based on observations by the local midwives and communication with the researchers, there are fewer admissions to the neonatal unit for birth asphyxia and fewer babies are being placed on the ‘dead baby’ bench.

### Limitations

Initially, eight champion midwives were trained. These early adopters have continued to lead in creating collective self-efficacy. However, it may not be possible to duplicate this at another site due to a lack of long-term relationships with the staff. Furthermore, not all midwives may learn effectively with high levels of arousal and stress, which may cause HOT resuscitation training to be ineffective.

## Conclusions

A champion programme must first have one or two committed mentors/champions. Creating champions can be a challenging process if only classroom-based education is available. The champion programme created confidence and mastery. The mantra of ‘air, air, air’ focuses trainees on ventilation in the classroom and at the bedside. Frequent labour ward training based on HOT resuscitation enhanced individual and collective self-efficacy among local midwives. Persistence, troubleshooting and real-life bedside teaching create an immediate feedback loop for the learners. Similar programs can empower midwifes to master new skills that can improve clinical delivery, instil hope and reduce VENDs in low-resource settings.

## Data Availability

The data supporting the findings of this study are available from the National Institute for Medical Research, Tanzania, but restrictions apply to the availability of these data, thus they are not publicly available. The data are available from the authors upon reasonable request and with permission from the National Institute for Medical Research.
